# Cerebrovascular Regulation in Neurological Disorders

**DOI:** 10.1155/2018/8140545

**Published:** 2018-10-08

**Authors:** Yi Yang, David Simpson, Bingren Hu, Jia Liu, Li Xiong

**Affiliations:** ^1^Department of Neurology, The First Hospital of Jilin University, Changchun, China; ^2^Institute of Sound and Vibration Research, University of Southampton, Southampton, UK; ^3^Shock Trauma and Anesthesiology Research Center, University of Maryland School of Medicine, Baltimore, MD, USA; ^4^Shenzhen Institutes of Advanced Technology, Chinese Academy of Sciences, Xueyuan Avenue, Shenzhen University Town, Shenzhen, China; ^5^Department of Medicine and Therapeutics, The Chinese University of Hong Kong, Prince of Wales Hospital, Hong Kong, China

Cerebrovascular regulation, referring to cerebral autoregulation, cerebrovascular reactivity, and neurovascular coupling, is intrinsic control mechanism of cerebral blood flow and is considered critical for the maintenance of adequate cerebral perfusion. Much previous work has related cerebrovascular regulation to the occurrence and prognosis of a range of neurological disorders, including cerebrovascular diseases, trauma, stroke, cognitive impairment, and neuropsychiatric disorders. Though many studies have identified these links, clinical exploitation of cerebrovascular regulation remains poorly developed. In addition, optimal methods for evaluation and data analysis in clinical studies need to be further discussed. In this special issue, we have brought together several papers that investigate cerebrovascular regulation in neurological disorders. We hope that this issue can encourage further studies on a number of important aspects of cerebrovascular regulation.

The study by J. M. D. van den Brule et al. entitled “Influence of Induced Blood Pressure Variability on the Assessment of Cerebral Autoregulation in Patients after Cardiac Arrest” addresses the influence of increased blood pressure variability on cerebral autoregulation assessment. Cerebral autoregulation measurements are performed in comatose patients after cardiac arrest both at rest and during intervention (tilting of bed). The review of J. M. D. van den Brule et al. entitled “Cerebral Perfusion and Cerebral Autoregulation after Cardiac Arrest” focuses on the alteration of cerebral perfusion and cerebral autoregulation after cardiac arrest, both of which are important in the development of secondary brain damage. The temporal course of cerebral blood flow after the return of spontaneous circulation, as well as cerebral autoregulation after cardiac arrest, is described in this review article.

X. Nie et al.'s review entitled “Futile Recanalization after Endovascular Therapy in Acute Ischemic Stroke” summarizes the predictors of futile recanalization and provides support for clinicians to make informed decisions about vascular recanalization therapy. Futile recanalization is one of the main causes of endovascular treatment failure and poor outcome. Impairment of cerebral blood flow regulation, bad collateral circulation, subacute reocclusion, large hypoperfusion volumes, and microvascular compromise were shown to be involved in this complicated process. The paper of Y. Ma et al. entitled “Pinocembrin Protects Blood-Brain Barrier Function and Expands the Therapeutic Time Window for Tissue-Type Plasminogen Activator Treatment in a Rat Thromboembolic Stroke Model” presents a study of the protective effects of pinocembrin on t-PA administration-induced blood-brain barrier damage in a rat thromboembolic stroke model. The potential mechanisms with which blood-brain barrier damage contributes to hemorrhagic transform after t-PA treatment are still unclear. The disruption of cerebrovascular regulation may play an important role in it.

M. L. Bøthun's study entitled “Time Course of Cerebrovascular Reactivity in Patients Treated for Unruptured Intracranial Aneurysms: A One-Year Transcranial Doppler and Acetazolamide Follow-Up Study” addresses the time course of cerebrovascular reactivity (CVR) in patients treated for unruptured intracranial aneurysms, by comparing CVR within the first week after aneurysm treatment with CVR one year later. Factors that influence stability of CVR over time are also proposed in this study. The study of S. Lv et al. entitled “Compromised Dynamic Cerebral Autoregulation in Patients with Epilepsy” investigates cerebral autoregulation capability in patients with epilepsy during the interictal period and explores factors related to cerebral autoregulation parameters. The possible mechanisms of compromised cerebral autoregulation are discussed. N.-F. Chi et al. present a study entitled “Comparing Different Recording Lengths of Dynamic Cerebral Autoregulation: 5 versus 10 Minutes”, in which dynamic cerebral autoregulation indices between 5- and 10-minute recordings in patients with ischemic stroke and healthy controls are compared. The study indicates that, to minimize the influences of time-dependent autoregulation variables, recording length should be unified in a single study or between studies.

The papers of this special issue provide new insights into cerebrovascular regulation in neurological disorders through its mechanisms, assessments, and clinical importance. Nevertheless, further studies are needed to help us to unlock the mystery of cerebrovascular regulation and its underlying mechanisms in neurological disorders, address technical challenges, and move on to providing the much-expected benefit to our patients.

